# Correlation of PKM2 and CD44 Protein Expression with Poor Prognosis in Platinum-Treated Epithelial Ovarian Cancer: A Retrospective Study

**DOI:** 10.3390/cancers12041013

**Published:** 2020-04-20

**Authors:** Chara Papadaki, Stavroula Manolakou, Eleni Lagoudaki, Spyros Pontikakis, Despo Ierodiakonou, Konstantinos Vogiatzoglou, Ippokratis Messaritakis, Maria Trypaki, Linda Giannikaki, Maria Sfakianaki, Antonia Kalykaki, Dimitrios Mavroudis, Maria Tzardi, John Souglakos

**Affiliations:** 1 Laboratory of Translational Oncology, Medical School, University of Crete, Heraklion, GR-71003 Crete, Greece; chapapadak@uoc.gr (C.P.); manolakoustauroula@gmail.com (S.M.); spontika@gmail.com (S.P.); vogiatzogloukwstas@gmail.com (K.V.); i_messaritakis@yahoo.com (I.M.); tr.maria@gmail.com (M.T.); mimasf19@gmail.com (M.S.); mavroudis@uoc.gr (D.M.); 2 Laboratory of Pathology, University General Hospital of Heraklion, GR-71003 Crete, Greece; elagoudakimd@gmail.com (E.L.); tzardi@med.uoc.gr (M.T.); 3 Health Planning Unit, Department of Social Medicine, Faculty of Medicine, University of Crete, Voutes Campus, Heraklion, GR-71003 Crete, Greece; desierod@gmail.com; 4 Laboratory of Pathology, Venizeleion General Hospital of Heraklion, GR-71409 Crete, Greece; lindagianikak@hotmail.com; 5 Department of Medical Oncology, University General Hospital of Heraklion, GR-71110 Crete, Greece; akalykaki@yahoo.gr

**Keywords:** CD44, PKM2, platinum-resistance, ovarian cancer

## Abstract

CD44, a surface marker for cancer stem cells, interacts with PKM2, a key regulator of aerobic glycolysis, and enhances the glycolytic phenotype of cancer cells leading to antioxidant protection and macromolecules’ synthesis. To clarify the clinical importance of this “cross-talk” as a mechanism of drug resistance, we assessed the expression both of PKM2 and of CD44 in cancer cells of patients with epithelial ovarian cancer (EOC) treated with platinum-based treatment. One hundred and seventy-one patients with EOC were assessed for PKM2mRNA expression and PKM2 and CD44 proteins detection. Associations with progression-free survival (PFS) and overall survival (OS) were assessed with Kaplan–Meier and adjusted Cox regression models. PKM2mRNA and protein as well as CD44 protein were detectable in the majority of patients. Positive correlation between PKM2 and CD44 protein expression was observed (Spearman rho = 0.2, *p* = 0.015). When we used the median to group patients into high versus low expression, high PKM2mRNA and protein levels were significantly associated with lower progression-free survival (PFS; *p* = 0.003 and *p* = 0.002, respectively) and shorter overall survival (OS; *p* ≤ 0.001 and *p* = 0.001, respectively). However, high CD44 protein expression was significantly correlated only with shorter OS (*p* = 0.004). Moreover, patients with both high PKM2 and CD44 protein levels experienced shorter PFS and OS (*p* = 0.007 and *p* = 0.003, respectively) compared to patients with low expression of both proteins. Finally, higher PKM2mRNA and protein expression as well as CD44 protein expression (HR: 2.16; HR: 1.82; HR: 1.01, respectively) were independent prognostic factors for decreased median OS (mOS), whereas only PKM2 protein expression (HR: 1.95) was an independent prognostic factor for decreased median PFS (mPFS). In conclusion, PKM2 expression is a negative prognostic factor in EOC patients, but the interaction between CD44 and PKM2 that may be implicated in EOC platinum-resistance needs further investigation.

## 1. Introduction

Ovarian cancer is the leading cause of death from gynecologic cancer in the developed world [1]. High mortality rates can be attributed to the absence of symptoms at early stage and to the fact that more than 70% of patients present with advanced stage at the time of diagnosis [2,3]. Epithelial ovarian cancer (EOC) is the most common type, comprising 90% of all types of ovarian cancer. The standard treatment for EOC patients is cytoreductive surgery followed by platinum-based chemotherapy. Carboplatin in combination with paclitaxel is the standard of care that can improve overall survival of patients with previously untreated advanced ovarian cancer [4,5,6,7]. Despite advances made in first-line treatment options, the majority of patients will eventually relapse and develop resistance to platinum-based chemotherapy [2,8].

A number of resistance mechanisms have been described in vitro; however, advances made in molecular profiling techniques have improved our understanding of drug resistance in tumors [9]. Mechanisms of tumor resistance to platinum agents are multi-factorial and may include decreased drug accumulation, increased levels of glutathione and metallothiones that contribute to drug detoxification, and enhanced DNA repair, which can remove platinum adducts from DNA [10,11].

PKM2, the M2 isoform of pyruvate kinase, is preferentially expressed in tumor cells and has been identified as a major regulator of aerobic glycolysis or “Warburg effect” [12,13,14], which is considered as a hallmark of cancer [15]. PKM2 has lower enzymatic activity in tumor cells compared to other pyruvate kinase isoforms [16,17], leading to an accumulation of glucose intermediates needed for tumor growth [18,19]. 

In the last decade, PKM2 has attracted an increased interest not only due to its regulatory role in abnormal cancer cell metabolism, but also due to its multiple non-metabolic functions. PKM2 has been shown to translocate from the cytoplasm to the nucleus acting as a transcriptional co-activator and as a protein kinase, suggesting diverse roles during tumorigenesis and cancer cell proliferation [20,21,22,23,24,25]. Recently, in vitro results suggested that PKM2 interacts with CD44, a cell surface marker for cancer stem cells and enhances the glycolytic phenotype of tumor cells [26]. This interaction has been shown to downregulate the intracellular oxidative load and to increase the glutathione antioxidant protection, suggesting a potential role of PKM2 in drug resistance [27,28]. We have recently investigated PKM2 and its potential predictive role in the outcome of non-small cell lung cancer (NSCLC) patients treated with cisplatin in first line setting. Moreover, other in vitro studies have been demonstrated the crucial role of PKM2 in platinum-resistance [29,30,31]. 

With regard to these current results, the aim of the present study was to evaluate the feasibility of PKM2 expression analysis as a biomarker in EOC formalin-fixed paraffin-embedded (FFPE) samples by immunocytochemistry as well as by qRT-PCR. Furthermore, we investigated the recently suggested contribution of CD44 and PKM2 or/and their interaction to platinum resistance in EOC patients. 

## 2. Results

### 2.1. Patients’ Characteristics 

Initially, one hundred and eighty-seven specimens from patients with EOC were included in the study, but, finally, 171 EOC FFPE tumor samples were analyzed because only these patients had received front-line platinum-based treatment. To be more specific, one hundred and fifty-seven patients (91.8%) received carboplatin–paclitaxel combination, whereas 14 (8.2%) patients administered other platinum-based chemotherapy (either carboplatin monotherapy or cisplatin–carboplatin combination) in the first line setting. Table 1 summarizes the clinicopathological characteristics of the 171patients of this study. The median age of the patients was 60 years (range 28–84 years), 110 (64.3%) had serous histology, 92 (53.8%) were poorly differentiated, and 123 (71.9%) had stage III–IV. Optimal debulking was achieved to 48 (28.1%) patients and from 57 patients who underwent second-look laparotomy, the majority of them had macroscopic partial response (17.5%). Moreover 46.2% of patients had platinum-sensitive disease. Median PFS was 11.4 months (95%Cl 9.2–13.5), whereas median OS was 43.1 months (95%Cl 34.9–51.4).

### 2.2. Gene and Protein Expression

Of the 171 EOC FFPE primary tumors analyzed, 159 specimens were available for PKM2 mRNA assessment, 171 specimens for PKM2, and 171 for CD44 protein expression analysis. Median value for PKM2 mRNA score was 25.55 rqV (range 2.17–81.56) and was used as a cut-off to classify patients as high (above or equal to cut-off) or low (below cut-off) expression. Low tumoral PKM2 mRNA expression was assessed in 72 (42.1%) patients, whereas 73 (42.7%) patients had high PKM2 mRNA levels.

**Figure 1:**
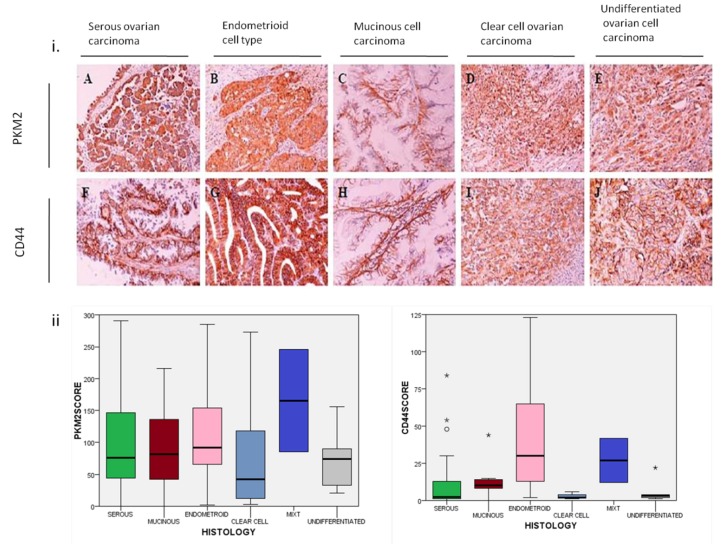
(**i**) Immunohistochemical (IHC) representative staining of PKM2 in the cytoplasm and of CD44 mainly in cell membranous and in cytoplasm in different histological subtypes of EOC tumor tissues (magnification 200×). (**A**) PKM2 and (**F**) CD44 staining in serous ovarian carcinoma. (**B**) PKM2 and (**G**) CD44 staining in endometroid cell type. (**C**) PKM2 and (**H**) CD44 protein expression in mucinous cell carcinoma. (**D**) PKM2 and (**I**) CD44 in clear cell ovarian carcinoma. (**E**) PKM2 and (**J**) CD44 expression in undifferentiated ovarian cell carcinoma. (**ii**) Boxplots showing PKM2 and CD44 protein expression distribution by different histology subtypes of ovarian carcinoma specimens (Wilcoxon rank test *p*-value = 0.004 for CD44 and *p*-value = 0.540 for PKM2 protein expression). PKM2: pyruvate kinase M2; EOC: epithelial ovarian cancer; *: extreme outliers.

**Figure 2:**
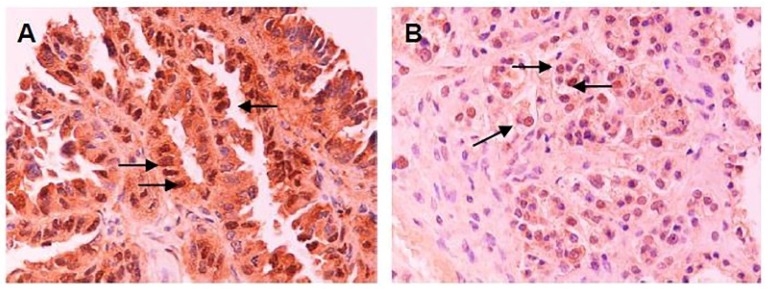
Nuclear immunoperoxidase staining of PKM2 (arrows) in serous (**A**) and clear cell (**B**) ovarian carcinoma (200×).

PKM2 cytoplasmic immunostaining was detectable in 167 out of 171 (97%) specimens, whereas CD44 membranous staining in 148 out of 172 (86%) tumor samples (Figure 1). Median histoscore for PKM2 protein expression was assessed to 101 (range 0–294) and to 18 (range 0–258) for CD44 protein expression. Using median scores as cut-off values, low PKM2 protein expression was observed in 100 (58.5%) patients and high expression in 71 (41.5%) patients. Similarly, 85 (49.7%) patients had low and 86 (50.3%) had high CD44 protein expression. Furthermore, nuclear PKM2 staining was observed in 10 (5.8%) patients’ tumor samples (Figure 2). 

No significant correlation was observed between PKM2 mRNA score and PKM2 histoscore (Spearman’s rho = 0.076; *p* = 0.362). However, a positive correlation was observed between PKM2 and CD44 histoscores (Spearman’s test = 0.185; *p* = 0.015).

### 2.3. Patients’ Outcome according to Expression

**Figure 3:**
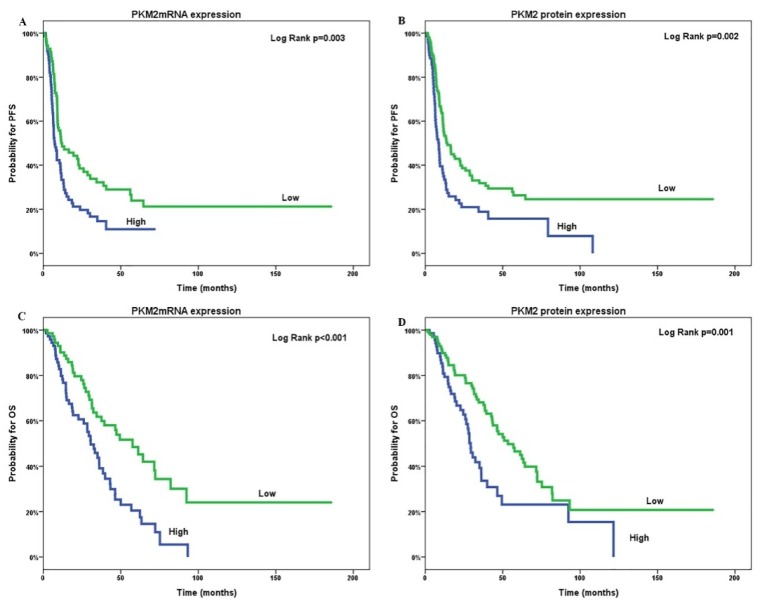
Tumoral PKM2mRNA (**A**,**C**) and protein expression (**B**,**D**) associated with progression-free survival (PFS; **A**,**B**) and overall survival (OS; **C**,**D**) of patients with epithelial ovarian cancer. PFS: progression-free survival; OS: overall survival.

**Figure 4:**
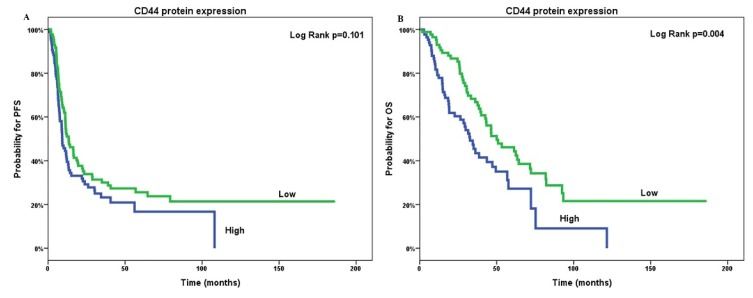
Correlation of tumoral CD44 protein expression (**A**,**B**) with overall survival (OS; **A**) and progression-free survival (PFS; **B**) of patients with epithelial ovarian cancer. OS: overall survival; PFS: progression-free survival.

Patients with high tumoral PKM2 mRNA and protein expression were associated with shorter PFS (medianPFS (mPFS); 7.3 versus 12.2 months; *p* = 0.003 and 8.7 versus 14.2 months; *p* = 0.002, respectively; Figure 3a,b) compared to patients with low expression. Similarly, shorter OS was significantly correlated with high tumoral PKM2 mRNA and protein expression (medianOS (mOS); 30.6 versus 57.6 months; *p* ≤ 0.001 and 29.3 versus 53.3 months; *p* = 0.001, respectively; Figure 3c,d). Furthermore, patients with high CD44 protein expression had significantly decreased OS (mOS; 32.6 versus 50 months; *p* = 0.004; Figure 4a) compared to those with low CD44 protein levels. In contrast, no significant correlation was observed between PFS and CD44 protein expression (*p* = 0.101; Figure 4b).

**Figure 5:**
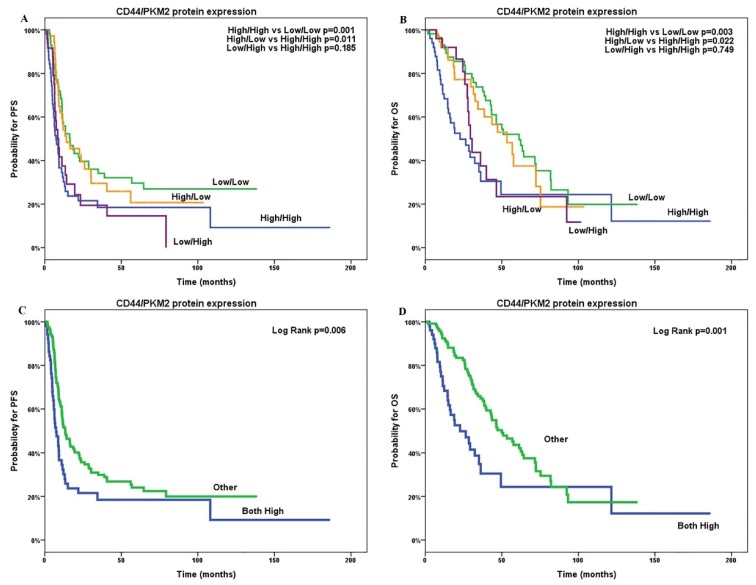
Combinations of CD44 and PKM2 protein co-expression and progression-free survival (PFS; **A**,**C**) and overall survival (OS; **B**,**D**) in patients with epithelial ovarian cancer. PFS: progression-free survival; OS: overall survival.

To further investigate the role of PKM2 and CD44 tumoral expression on patients’ outcome, we analyzed patients according to the combined expression for both PKM2 and CD44. Patients with low PKM2/CD44 protein expression levels experienced significantly increased PFS (mPFS; 16.5 versus 7.4; *p* = 0.003, Figure 5a) and OS (mOS; 61.3 versus 22.7; *p* = 0.001, Figure 5b) compared with patients with high PKM2/CD44 tumoral expression. In addition, patients with PKM2 high/CD44 low expression appeared with increased PFS and OS compared with PKM2 high/CD44 high patients (mOS8, 5 versus 7.4; *p* = 0.749 and 30.6 versus 22.7; *p* = 0.185) but without statistically significance (Table 2). However, CD44 high/PKM2 low patients showed significantly better PFS and OS compared with PKM2 high/CD44 high group. 

### 2.4. Multivariate and Univariate Analysis

Univariate Cox regression analyses demonstrated that poorly-differentiated tumors (HR: 1.73, 95%CI: 1.21–2.50; *p* = 0.003), advanced stage (III–IV; HR: 1.07, 95%CI: 1.08–2.59; *p* = 0.022), platinum-resistance (HR:4.12, 95%CI: 2.58–2.56; *p* < 0.001) as well as residual tumor after primary surgery (HR: 2.15, 95%CI: 1.39–3.332; *p* = 0.001)were significantly associated with PFS (Table 3). However, no significant associations were revealed between older age (*p* = 0.896) or histology (*p* = 0.495) and PFS. High PKM2 mRNA levels (HR: 1.74, 95%CI: 1.20–2.54; *p* = 0.004) had significant association with PFS, whereas no statistically significant association between PKM2 mRNA score and PFS (*p* = 0.253) were shown. Nevertheless, higher PKM2 protein levels associated with shorter PFS (HR: 1.002, 95% CI: 1.001–1.004, *p* = 0.043; for high versus low HR: 1.74, 95%CI: 1.22–2.48; *p* = 0.002). Finally, CD44 protein expression was significantly associated with PFS (HR: 1.01, 95%CI: 1.00–1.01; *p* = 0.007); this association did not remain significant when a median cut-off was used (i.e., high versus low; *p* = 0.102).Regarding OS, platinum-resistance(HR: 4.03, 95%CI: 2.43–6.68; *p* < 0.001) and residual tumor after primary surgery (HR: 1.72, 95%CI: 1.07–2.75; *p* = 0.024) were significantly associated with OS, but OS was not associated with older age (*p* = 0.077), histology (*p* = 0.070), poor differentiation (*p* = 0.073), or advanced stage (III–IV; *p* = 0.084) (Table 3). High PKM2 mRNA expression levels (HR: 2.15, 95%CI: 1.39–3.32; *p* = 0.001) were significantly associated with OS, but not when PKM2 mRNA was used continuously. Higher PKM2 protein levels were significantly associated with OS (HR: 1.0, 95%CI: 1.00–1.00; *p* = 0.05; high versus low HR: 1.94, 95%CI: 1.28–2.94; *p* = 0.002), as well as higher CD44 protein levels (HR: 1.01, 95%CI: 1.00–1.01; *p* = 0.018, high versus low HR: 1.80, 95%CI: 1.20–2.70; *p* = 0.005). When combined together, with both proteins being high, the risk for PFS and OS increased (HR: 1.69, 95%CI: 1.16–2.47 and HR: 2.13, 95%CI: 1.38–3.30, respectively).

When adjusting the models for significant covariates of univariate analysis, PFS was significantly associated with high PKM2 (HR: 1.95, 95%CI: 1.23–3.09; *p* = 0.004), and PKM2 high/CD44 high protein expression (HR: 1.66, 95%CI: 1.03–2.7; *p* = 0.04), while OS was significantly associated with both high PKM2 mRNA (HR: 2.16, 95%CI: 1.07–4.34; *p* = 0.034), and protein expression (HR: 1.82, 95%CI: 1.05–3.17; *p* = 0.034) as well as with increase in CD44 protein expression (HR: 1.01, 95%CI: 1.00–1.01; *p* = 0.033). Combined PKM2 high and CD44 high protein expression was also associated with worse OS (HR: 1.99, 95%CI: 1.10–3.60; *p* = 0.023). Other independent prognostic factors for PFS were also poorly-differentiated tumors (HR: 1.73, 95%CI: 1.07–2.82; *p* = 0.026), and platinum-resistant tumors (HR: 3.17, 95%CI: 1.95–5.14; *p* < 0.001) and for OS were residual tumor after primary surgery (HR: 3.60, 95%CI: 1.41–9.21; *p* = 0.008), as well (Table 3).

## 3. Discussion

Exposure of cancer cells to chemotherapy results in alteration of the intracellular metabolic status and increase of oxidant load that in turn leads to fatal damage of the vital macromolecules. The chemosensitivity is variable among the several type of tumors or among the different cells of the same tumor. In contrast the phenomenon of chemoresistance, either acquired or intrinsic, may lead to failure of cancer treatment [32]. In advanced EOC, the majority of the patients relapse, being platinum-refractory or resistant, but the exact mechanisms are still yet under investigation. 

In this study, PKM2 expression was assessed as prognostic and predictive biomarker to platinum-based treatment in EOC patients. Indeed, there are several in vitro studies that demonstrate the interference of aerobic glycolysis and of PKM2 on platinum-sensitivity by modification of oxidative phosphorylation rate, by alteration of lactate production, and by restriction of drug oxidative role. For instance, knockdown of PKM2 expression in colorectal and bladder cancer cells re-boost platinum sensitivity and induce cell apoptosis [33,34]. Another recent study performed in ovarian cancer cell lines showed the overexpression of PKM2 that in turn increased CCND1 expression, whereas it decreased CDKN1A expression; determinants involved in the increase of proliferation and in the decrease of apoptosis of ovarian cancer cells [35]. Moreover, PKM2 strong expression has been associated with poor response to chemotherapy in patients with esophageal squamous cell carcinoma [36]. 

In this retrospective study, interestingly, the high PKM2 mRNA and protein expression was associated with platinum-resistance in EOC. PKM2 protein expression was also proven that is independent prognostic factor for both mPFS and mOS for advanced EOC, evidence that may have clinical use to divide EOC patients in high versus poor prognostic groups and in turn to modify physicians’ treatment plans (i.e., platinum-based treatment versus anthracyclines, gemcitabine, or taxanes).

Additionally, the majority of FFPE specimens of this study had cytoplasmic PKM2 staining, but in few specimens nuclear localization of PKM2 was revealed as well. This finding confirms the pleiotropic activity of PKM2 that includes not only its enzyme role but also its role as a co-activator transcription factor [37,38]. More experiments are needed in order to detect the exact interactions of PKM2 transcription co-factor and the genes whereby the latter alters their transcription.

On the other hand, several studies have demonstrated the ability of cancer cells to modify the extracellular matrix synthesis and to gain advantage from its role in tumor progression. Not only is tumor growth induced by ECM remodeling, but also tumor migration and cellular differentiation [39]. Ovarian cancer cells are believed to disseminate by altering ECM synthesis and, in turn, migrateto neighbor organs or attach the peritoneum. Specifically, the hyaluronan receptor, called CD44, the hyaluronan (HA) itself, and hyaluronidase have been already investigated for their effect on EOC [40]. The former mediates intra-abdominal migration via binding peritoneal mesothelium and regulating epithelial–mesenchymal transition, interacts with other surface proteins (i.e., HER2), and its inhibition has been correlated with reduced risk of metastasis or local relapse [40,41]. However, there are up to ten different CD44 isoforms and their cross-talks with other intracellular signaling pathways have not yet fully characterized [42]. 

Here, we investigated the CD44 mRNA and protein expression on EOC specimens as well as CD44 interaction with the central regulator of aerobic glycolysis, PKM2. To our knowledge, few data are available in the literature on the correlation between adhesion and glycolytic proteins and its impact on platinum-resistance and survival of EOC patients. In this study, although high PKM2 mRNA and protein levels were associated with both reduced mPFS and mOS, high CD44 protein levels were associated only with reduced mOS. Similarly, in recent systematic analysis of 1201 patients with advanced carcinomas selected from fifteen studies, CD44 expression was linked only to worse OS and not to DFS, RFS, or PFS [43]. Nevertheless, the CD44 histoscore in our study, that is the continuous variable, not only was negatively associated with both mPFS and mOS but also was an independent prognostic factor for mOS in contrast with the PKM2 histoscore. This finding may indicate only a binary effect of PKM2 mRNA and protein expression on PFS and OS.

Notwithstanding, in our study the combination of high expression of PKM2 and CD44 proteins (“PKM2 high/CD44 high” status) had significant negative correlation with both mPFS and mOS, indicating a possible synergistic role of PKM2/CD44 in platinum-resistance of EOC cells. Indeed, CD44 expression has already been reported to not only to promote the metabolic shift of cancer cells via HIF-1 but also to induce glycolytic and antioxidant pathways via PKM2 in p53-mt/ hypoxic colorectal cell lines, leading to chemoresistance [26,28]. However, in this study CD44low/PKM2 high patients had increased mPFS and mOS, in contrast with PKM2 high/ CD44 high patients, but this difference was not statistically significant. Thus, more studies should be performed to elucidate the above mentioned hypothesis in respect with a possible synergistic effect of PKM2/CD44 or a possible interaction among them.

A limitation of the present study was the lack of prospective assessment of the prognostic value of PKM2 mRNA and PKM2/CD44 protein expression in EOC patients treated with platinum-based treatment. Furthermore, more mechanistic studies are necessary to assess PKM2 allosteric transformation and its effect on platinum-resistance [44]. Our ongoing studies will address the latter issue as well as the possible modulators of PKM2 allosterism in cancer cells, including the CD44 adhesion protein.

## 4. Materials and Methods

### 4.1. Patient’s Population

One hundred and seventy-two consecutive cases of women with newly diagnosed advanced epithelial ovarian cancer were retrospectively collected and analyzed. Followed cytoreductive surgery, all patients received platinum-based front line chemotherapy in the context of a multicenter phase III randomized trial conducted from the Hellenic Cooperative Oncology Group (HeCOG) [45,46]. Tumor staging (I–IV) was evaluated based on the standards of the FIGO (stage I: carcinoma limited to the vaginal wall; stage II: carcinoma involved the subvaginal tissue but not extended to the pelvic wall; stage III: carcinoma extended to the pelvic wall; stage IV: carcinoma extended beyond the true pelvis or involved the mucosa of the bladder or rectum) [46]. Clinicopathologic patients’ characteristics are summarized in Table 1.

### 4.2. Ethics Approval and Consent to Participate

The study was approved by the Ethics Committee/Institutional review board of the University Hospital of Heraklion (14753/01/12/2011) and signed informed consent was obtained from all enrolled patients. All procedures performed were in accordance with the ethical standards of the institutional and/or national research committee and with the 1964 Helsinki Declaration and its later amendments or comparable ethical standards.

### 4.3. Immunohistochemistry and Staining Evaluation

EOC 4μmthick FFPE tissue sections were stained with hematoxylin and eosin (H&N) and histopathologically verified by a pathologist. The expression of PKM2 and CD44 was determined immunohistochemically. Primary antibody used for PKM2 staining was against the specific sequence of exon 9 that is unique to PKM2 (rabbit polyclonal Ab, Cat No 3198, Cell Signaling; dilution 1:600). Primary antibody used for CD44 recognizes all forms of CD44 (mouse monoclonal Ab, clone G44-26, Cat No 550392, BD Pharmingen; dilution 1:600). Immunostaining was performed using Ultra Vision LP Quanto Detection System HRP Polymer (Thermo Fisher Scientific, Fremont, CA, USA). Sections stained for PKM2 were firstly treated in Tris/EDTA buffer (pH 9.0) for 13 min, whereas sections stained for CD44 were heated in a microwave oven in 0.01 M citrate buffer (pH 6.0). Slides were counterstained with hematoxylin. Staining evaluation was performed by two independent pathologists blinded to each other’s scores and to each patient’s clinical information. The intensity of PKM2 and CD44 staining was scored on a 0to3 scale, 0—negative, 1—light, 2—moderate, and 3—strong, as previously described. The percentage of the cells stained at each intensity was calculated by dividing the number of cells positive for the marker at each intensity by the total number of cells. Areas that were negative were given a value of 0. The entire tissue section was examined microscopically at high-power field (HPF) magnifications. The final histoscore (HSCORE) was calculated using the formula: [(1 × percentage of weakly positive tumor cells) + (2 × percentage of moderately positive tumor cells) + (3 × percentage of intense positive tumor cells)]. The median values of histoscores were used to classify samples as high (above or equal the median) or low (below the median) expression [47].

### 4.4. mRNA Expression Analysis

For RNA extraction, an Eppendorf piezoelectric microdissector (Eppendorf, Hamburg, Germany) was used to procure only malignant cells from tumor specimens. Trizol LS (Invitrogen, Carlsbad, CA, USA) was used for RNA extraction, followed by DNase (DNA- free, Ambion, Austin, TX, USA) treatment in order to avoid genomic DNA contamination, as previously described [48]. cDNA synthesis was then performed with 200 ng of total RNA using Superscript III reverse transcriptase (Invitrogen, Carlsbad, CA, USA). Relative cDNA quantification for *PKM2* and *β-actin* and *phosphoglycerate kinase 1* (*PGK*), as internal controls, was performed using the ABI Prism 7900 HT Sequence Detection System (Applied Biosystems, Foster city, CA, USA). The primers and probe sequences used for qRT-PCR have been reported elsewhere [49].

The primers and 5′-labeled fluorescent reporter dye (6FAM) probe sets were designed using the Primer Express 2.0 Software (Applied Biosystems, Zug, Switzerland) according to the Ref Seq NM_002654 for *PKM2* and were as follows: *PKM2*, 5′-GCCATAATCGTCCTCACCAAGT-3′ (forward), 5′-GCACGTGGGCGGTATCTG-3′ (reverse) and 5′-CAGGTCTGCTCACCAGG-3′ (probe). The primers and probe sequences for both housekeeping genes, *β-actin* and *PGK* have been reported elsewhere [50]. Comparative Ct method was used for gene expression analysis using both *β-actin* and *PGK* as reference genes and commercial RNA (STRATAGENE) as calibrators. Final expression values were determined as follows: 2−(ΔCT sample −ΔCT calibrator), where ΔCT values of the calibrator and sample were determined by subtracting the CT value of the target gene from the mean value of both reference genes. In all experiments, only triplicates with a SD of the CT value < 0.25 were accepted. In addition, genomic DNA contamination was excluded by including non-reverse transcribed RNA as a control for each sample.

### 4.5. Statistical Analysis

Baseline characteristics are presented as count (*n*) and percentage (%), and continuous non parametric data as median and range or 95% confidence interval (95%Cl). Correlations were tested with Spearman’s rho and group differences with non-parametric tests (e.g., Wilcoxon rank test). The cut-off point for mRNA and protein expression was defined according to the median values. Samples with expression above or equal to the median were considered as samples with high expression, while those with value below the median as samples with low expression. PFS and OS were defined as the time from treatment start to the first documented disease progression and death, respectively. Kaplan–Meier method was used to estimate the PFS and OS. Log-rank test was used to assess the differences in survival function between patients with high and low expression. Cox regression models was used to test for associations and provide hazard ratio (HR) and 95%CI of PFS and OS with PMK2 mRNA and protein and CD44 protein expression. With univariate cox regression, we assessed associations of PFS and OS with age and tumor characteristics, including age (>70 years versus <70years), histology (serous versus others), grade (poorly versus well-moderate), stage (III–IV versus I–II), platinum-sensitivity (resistant versus sensitive) and post-operation residual tumor, and those found significant were included in adjusted analyses as potential confounders.

All reported *p*-values are two sided with *p* < 0.05 considered as statistically significant. Statistical analysis was performed with IBM-SPSS software (IBM SPSS Statistics 20). All the laboratory analysis was performed blinding to the clinical data.

## 5. Conclusions

To our knowledge, this is the first attempt to demonstrate that high expression of CD44 adhesion glycoprotein and of PKM2 glycolytic enzyme was linked to unfavorable OS in EOC patients treated with platinum-based treatment. Moreover, detection of PKM2 protein expression is suggested as a novel prognostic biomarker that may possibly be used for division of EOC patients into high or low risk groups for platinum-resistance. However, the hypothesis of impact of both CD44 and PKM2 on the prognosis of EOC patients have not been yet elucidated. In addition, the exact mechanism of how CD44 perturbs the metabolic phenotype of EOC cells via its interaction with PKM2 remains obscure. More studies should be performed to discover if the above proteins may be appropriate to be used as novel diagnostic tools and/or therapeutic targets.

## Figures and Tables

**Table 1:** Patients’ and tumors’ characteristics.

Feature	*Ν*	%
Selected patients	171/186	91.5
Regimen		
Carboplatin–paclitaxel	157	91.8
Other platinum-based treatment	14	8.2
Median age (range) years	60 (28–84)	
≤70 years	145	84.8
>70 years	26	15.2
Histology		
Serous	110	64.3
Mucinous	15	8.8
Endometrioid	19	11.1
Clear cell	7	4.1
Other epithelial ^1^	20	11.7
Grade		
Well differentiated	15	8.8
Moderate	64	37.4
Poorly differentiated	92	53.8
Stage		
I	26	15.2
II	22	12.9
III	104	60.8
IV	19	11.1
Surgery		
Optimal	48	28.1
Others	121	70.8
Unknown	2	1.2
Second-look laparotomy (SLL)		
PCR ^2^	12	7.0
PPR Micro ^3^	15	8.8
PPR Macrp ^4^	30	17.5
Without SLL/unknown	116	66.7
Platinum-sensitivity		
YES	79	46.2
NO	37	21.6
Unknown	55	32.2
Median survival (range) months		
mPFS (95%CI) ^5^	11.4 (95%Cl 9.2–13.5)	
mOS (95%CI) ^6^	43.1 (95%Cl 34.9–51.4)	

^1^ Undifferentiated, unclassified, mixed, unknown; ^2^ pathological complete response; ^3^ microscopic partial response; ^4^ macroscopic partial response; ^5^ median progression-free survival; ^6^ median overall survival.

**Table 2:** Tumoral expression of PKM2 and CD44 and patients’ outcome.

Variables	*N*	PFS (Months)	*p*	OS (Months)	*p*
		Median (95%CI)		Median (95%CI)	
PKM2 mRNA expression					
Low	72	12.2 (2.4–21.9)	**0.003**	57.6 (40.0–75.2)	**<0.001**
High	73	7.3 (5.3–9.5)		30.6 (23.3–37.8)	
PKM2 protein expression					
Low	100	14.2 (8.3–20.1)	**0.002**	53.3 (37.8–68.9)	**0.001**
High	71	8.7 (6.7–10.7)		29.3 (26.0–32.6)	
CD44 protein expression					
Low	85	13.4 (8.5–18.2)	0.101	50.0 (31.89–68.1)	**0.004**
High	86	9.2 (7.0–11.4)		32.6 (26.0–39.2)	
PKM2/CD44 proteins’ co-expression					
PKM2Low/CD44 Low	57	16.5 (9.2–23.8)	**Ref.**	61.3 (41.6–80.9)	**Ref.**
PKM2 High/CD44 High	51	7.4 (4.6–10.1)	**0.003^1^**	22.7 (9.4–36.1)	**0.001^1^**
PKM2 Low/CD44 High	38	14.2 (1.6–26.8)	0.700^2^	53.3 (35.6–71.1)	0.614^2^
PKM2 High/CD44 Low	25	8.5 (5.7–11.2)	**0.031^3^**	30.6 (26.5–34.6)	0.154^3^
PKM2 High/CD44 High	51	7.4 (4.6–10.1)	**Ref.**	22.7 (9.4–36.1)	**Ref.**
PKM2 Low/CD44 Low	57	16.5 (9.2–23.8)	**0.003^4^**	61.3 (41.6–80.9)	**0.001^4^**
PKM2 Low/CD44 High	38	14.2 (1.6–26.8)	**0.022^5^**	53.3 (35.6–71.1)	**0.011^5^**
PKM2 High/CD44 Low	25	8.5 (5.7–11.2)	0.749**^6^**	30.6 (26.5–34.6)	0.185**^6^**

Bold-faceted *p*-values indicate statistically significant association (*p*-value < 0.05).

**Table 3:** Univariate and multivariate analysis for PFS and OS of EOC patients treated with platinum-based chemotherapy.

Variables	Unadjusted Hazard Ratio	95%CI	*p*-Value	Adjusted Hazard Ratio	95%CI	*p*-Value
**Progression-free survival**						
Age (>70 years versus <70 years)	1.03	0.60–1.60	0.896	-	-	-
Histology (serous versus others)	1.13	0.76–1.50	0.495	-	-	-
Grade (poorly versus well-moderate)	1.73	1.21–2.50	**0.003**	1.73	1.07–2.82	**0.026**
Stage (III–IV versus I–II)	1.67	1.08–2.59	**0.022**	1.30	0.57–2.93	0.527
Platinum-sensitivity (resistant versus sensitive)	4.12	2.58–6.56	**<0.001**	3.17	1.95–5.14	**<0.001**
Post-operation residual tumor	2.15	1.39–3.32	**0.001**	2.36	0.97–5.75	0.058
PKM2 mRNA	0.99	0.99–1.02	0.253	0.98	0.97–1.003	0.984
PKM2 mRNA high versus low	1.74	1.20–2.54	**0.004**	1.18	0.67–2.09	0.567
PKM2 protein	1.00	1.000–1.004	**0.043**	1.002	0.999–1.005	0.234
PKM2 protein high versus low	1.74	1.22–2.48	**0.002**	1.95	1.23–3.09	**0.004**
CD44 protein	1.01	1.002–1.011	**0.007**	1.01	0.999–1.005	0.090
CD44 protein high versus low	1.34	0.94–1.90	0.102	1.21	0.77–1.88	0.409
CD44/PKM2 protein (both high versus others)	1.69	1.16–2.47	**0.006**	1.66	1.03–2.7	**0.040**
**OverallSurvival**						
Age (>70years versus <70years)	1.09	0.63–1.89	0.770	-	-	-
Histology (serous versus others)	1.45	0.97–2.18	0.070	-	-	-
Grade (poorly versus well-moderate)	1.45	0.97–2.18	0.073	-	-	-
Stage (III–IV versus I–II)	1.57	0.94–2.62	0.084	-	-	-
Platinum-sensitivity (resistant versus sensitive)	4.03	2.43–6.68	**<0.001**	3.11	1.85–5.21	**<0.001**
Post-operation residual tumor	1.72	1.07–2.75	**0.024**	3.60	1.41–9.21	**0.008**
PKM2 mRNA high versus low	2.15	1.39–3.32	**0.001**	2.16	1.07–4.34	**0.034**
PKM2 mRNAscore	1.00	0.99–1.02	0.899	0.998	0.999–1.020	0.871
PKM2 protein	1.002	1.000–1.005	**0.050**	1.003	0.999–1.006	0.177
PKM2 protein high versus low	1.94	1.28–2.94	**0.002**	1.82	1.05–3.17	**0.034**
CD44 protein	1.006	1.006–1.011	**0.018**	1.01	1.00–1.01	**0.033**
CD44 protein (high versus low)	1.80	1.20–2.70	**0.005**	1.61	0.97–2.68	0.065
CD44/PKM2 protein (bothhigh versus others)	2.13	1.38–3.30	**0.001**	1.99	1.10–3.60	**0.023**

Bold-faceted *p*-values indicate statistically significant association (*p*-value < 0.05).

## References

[1] Siegel R., Naishadham D., Jemal A. (2012). Cancer statistics, 2012. CA Cancer J. Clin..

[2] Cannistra S.A. (2004). Cancer of the ovary. N. Engl. J. Med..

[3] Heintz A.P., Odicino F., Maisonneuve P., Quinn M.A., Benedet J.L., Creasman W.T., Ngan H.Y., Pecorelli S., Beller U. (2006). Carcinoma of the ovary. FIGO 26th Annual Report on the Results of Treatment in Gynecological Cancer. Int. J. Gynaecol. Obstet..

[4] Du Bois A., Lück H.J., Meier W., Adams H.P., Möbus V., Costa S., Bauknecht T., Richter B., Warm M., Schröder W. (2003). A randomized clinical trial of cisplatin/paclitaxel versus carboplatin/paclitaxel as first-line treatment of ovarian cancer. J. Natl. Cancer Inst..

[5] Ozols R.F., Markman M., Thigpen J.T. (2002). ICON3 and chemotherapy for ovarian cancer. Lancet.

[6] McGuire W.P., Hoskins W.J., Brady M.F., Kucera P.R., Partridge E.E., Look K.Y., Clarke-Pearson D.L., Davidson M. (1996). Cyclophosphamide and cisplatin compared with paclitaxel and cisplatin in patients with stage III and stage IV ovarian cancer. N. Engl. J. Med..

[7] Piccart M.J., Bertelsen K., James K., Cassidy J., Mangioni C., Simonsen E., Stuart G., Kaye S., Vergote I., Blom R. (2000). Randomized intergroup trial of cisplatin-paclitaxel versus cisplatin-cyclophosphamide in women with advanced epithelial ovarian cancer: Three-year results. J. Natl. Cancer Inst..

[8] Pfisterer J., Ledermann J.A. (2006). Management of platinum-sensitive recurrent ovarian cancer. Semin. Oncol..

[9] Agarwal R., Kaye S.B. (2003). Ovarian cancer: Strategies for overcoming resistance to chemotherapy. Nat. Rev. Cancer.

[10] Kelland L. (2007). The resurgence of platinum-based cancer chemotherapy. Nat. Rev. Cancer.

[11] Read T.E., Kodner I.J. (1999). Colorectal cancer: Risk factors and recommendations for early detection. Am. Fam. Phys..

[12] Christofk H.R., Vander Heiden M.G., Harris M.H., Ramanathan A., Gerszten R.E., Wei R., Fleming M.D., Schreiber S.L., Cantley L.C. (2008). The M2 splice isoform of pyruvate kinase is important for cancer metabolism and tumour growth. Nature.

[13] Warburg O. (1956). Origin of cancer cells. Oncologia.

[14] Warburg O. (1956). On the origin of cancer cells. Science.

[15] Hanahan D., Weinberg R.A. (2011). Hallmarks of cancer: The next generation. Cell.

[16] Christofk H.R., Vander Heiden M.G., Wu N., Asara J.M., Cantley L.C. (2008). Pyruvate kinase M2 is a phosphotyrosine-binding protein. Nature.

[17] Anastasiou D., Yu Y., Israelsen W.J., Jiang J.K., Boxer M.B., Hong B.S., Tempel W., Dimov S., Shen M., Jha A. (2012). Pyruvate kinase M2 activators promote tetramer formation and suppress tumorigenesis. Nat. Chem. Biol..

[18] Jiang L., Deberardinis R.J. (2012). Cancer metabolism: When more is less. Nature.

[19] Mazurek S., Boschek C.B., Hugo F., Eigenbrodt E. (2005). Pyruvate kinase type M2 and its role in tumor growth and spreading. Semin Cancer Biol..

[20] Keller K.E., Doctor Z.M., Dwyer Z.W., Lee Y.S. (2014). SAICAR induces protein kinase activity of PKM2 that is necessary for sustained proliferative signaling of cancer cells. Mol. Cell.

[21] Gao X., Wang H., Yang J.J., Liu X., Liu Z.R. (2012). Pyruvate kinase M2 regulates gene transcription by acting as a protein kinase. Mol. Cell.

[22] Luo W., Hu H., Chang R., Zhong J., Knabel M., O’Meally R., Cole R.N., Pandey A., Semenza G.L. (2011). Pyruvate kinase M2 is a PHD3-stimulated coactivator for hypoxia-inducible factor 1. Cell.

[23] Yang W., Xia Y., Hawke D., Li X., Liang J., Xing D., Aldape K., Hunter T., Yung W.K.A., Lu Z. (2014). PKM2 Phosphorylates Histone H3 and Promotes Gene Transcription and Tumorigenesis. Cell.

[24] Yang W., Xia Y., Ji. H., Zheng Y., Liang J., Huang W., Gao X., Aldape K., Lu Z. (2017). Corrigendum: Nuclear PKM2 regulates beta-catenin transactivation upon EGFR activation. Nature.

[25] Yang W., Zheng Y., Xia Y., Ji H., Chen X., Guo F., Lyssiotis C.A., Aldape K., Cantley L.C., Lu Z. (2012). ERK1/2-dependent phosphorylation and nuclear translocation of PKM2 promotes the Warburg effect. Nat. Cell Biol..

[26] Tamada M., Suematsu M., Saya H. (2012). Pyruvate kinase M2: Multiple faces for conferring benefits on cancer cells. Clin. Cancer Res..

[27] Anastasiou D., Poulogiannis G., Asara J.M., Boxer M.B., Jiang J.K., Shen M., Bellinger G., Sasaki A.T., Locasale J.W., Auld D.S. (2011). Inhibition of pyruvate kinase M2 by reactive oxygen species contributes to cellular antioxidant responses. Science.

[28] Tamada M., Nagano O., Tateyama S., Ohmura M., Yae T., Ishimoto T., Sugihara E., Onishi N., Yamamoto T., Yanagawa H. (2012). Modulation of glucose metabolism by CD44 contributes to antioxidant status and drug resistance in cancer cells. Cancer Res..

[29] Guo W., Zhang Y., Chen T., Wang Y., Xue J., Zhang Y., Xiao W., Mo X., Lu Y. (2011). Efficacy of RNAi targeting of pyruvate kinase M2 combined with cisplatin in a lung cancer model. J. Cancer Res. Clin. Oncol..

[30] Martinez-Balibrea E., Plasencia C., Ginés A., Martinez-Cardús A., Musulén E., Aguilera R., Manzano J.L., Neamati N., Abad A. (2009). A proteomic approach links decreased pyruvate kinase M2 expression to oxaliplatin resistance in patients with colorectal cancer and in human cell lines. Mol. Cancer Ther..

[31] Yoo B.C., Ku J.L., Hong S.H., Shin Y.K., Park S.Y., Kim H.K., Park J.G. (2004). Decreased pyruvate kinase M2 activity linked to cisplatin resistance in human gastric carcinoma cell lines. Int. J. Cancer.

[32] Zheng H.C. (2017). The molecular mechanisms of chemoresistance in cancers. Oncotarget.

[33] Lu W.Q., Hu Y.Y., Lin X.P., Fan W. (2017). Knockdown of PKM2 and GLS1 expression can significantly reverse oxaliplatin-resistance in colorectal cancer cells. Oncotarget.

[34] Zhu Q., Hong B., Zhang L., Wang J. (2018). Pyruvate kinase M2 inhibits the progression of bladder cancer by targeting MAKP pathway. J. Cancer Res. Ther..

[35] Zheng B., Liu F., Zeng L., Geng L., Ouyang X., Wang K., Huang Q. (2018). Overexpression of Pyruvate Kinase Type M2 (PKM2) Promotes Ovarian Cancer Cell Growth and Survival Via Regulation of Cell Cycle Progression Related with Upregulated CCND1 and Downregulated CDKN1A Expression. Med. Sci. Monit..

[36] Carinci F., Stabellini G., Calvitti M., Pelucchi S., Targa L., Farina A., Pezzetti F., Pastore A. (2002). CD44 as prognostic factor in oral and oropharyngeal squamous cell carcinoma. J. Craniofac. Surg..

[37] He X., Du S., Lei T., Li X., Liu Y., Wang H., Tong R., Wang Y. (2017). PKM2 in carcinogenesis and oncotherapy. Oncotarget.

[38] Hsu M.C., Hung W.C. (2018). Pyruvate kinase M2 fuels multiple aspects of cancer cells: From cellular metabolism, transcriptional regulation to extracellular signaling. Mol. Cancer.

[39] Chen C., Zhao S., Karnad A., Freeman J.W. (2018). The biology and role of CD44 in cancer progression: Therapeutic implications. J. Hematol. Oncol..

[40] Ween M.P., Oehler M.K., Ricciardelli C. (2011). Role of versican, hyaluronan and CD44 in ovarian cancer metastasis. Int. J. Mol. Sci..

[41] Nakamura K., Sawada K., Kinose Y., Yoshimura A., Toda A., Nakatsuka E., Hashimoto K., Mabuchi S., Morishige K.I., Kurachi H. (2017). Exosomes Promote Ovarian Cancer Cell Invasion through Transfer of CD44 to Peritoneal Mesothelial Cells. Mol. Cancer Res..

[42] Sacks J.D., Barbolina M.V. (2015). Expression and Function of CD44 in Epithelial Ovarian Carcinoma. Biomolecules.

[43] Han S., Huang T., Li W., Wang X., Wu X., Liu S., Yang W., Shi Q., Li H., Hou F. (2019). Prognostic Value of CD44 and Its Isoforms in Advanced Cancer: A Systematic Meta-Analysis with Trial Sequential Analysis. Front. Oncol..

[44] Macpherson J.A., Theisen A., Masino L., Fets L., Driscoll P.C., Encheva V., Snijders A.P., Martin S.R., Kleinjung J., Barran P.E. (2019). Functional cross-talk between allosteric effects of activating and inhibiting ligands underlies PKM2 regulation. Elife.

[45] Aravantinos G., Fountzilas G., Kosmidis P., Dimopoulos M.A., Stathopoulos G.P., Pavlidis N., Bafaloukos D., Papadimitriou C., Karpathios S., Georgoulias V. (2005). Paclitaxel plus carboplatin versus paclitaxel plus alternating carboplatin and cisplatin for initial treatment of advanced ovarian cancer: Long-term efficacy results: A Hellenic Cooperative Oncology Group (HeCOG) study. Ann. Oncol..

[46] FIGO Committee on Gynecologic Oncology (2009). Current FIGO staging for cancer of the vagina, fallopian tube, ovary, and gestational trophoblastic neoplasia. Int. J. Gynaecol. Obstet..

[47] McCarty K.S.J., Szabo E., Flowers J.L., Cox E.B., Leight G.S., Miller L., Konrath J., Soper J.T., Budwit D.A., Creasman W.T. (1986). Use of a Monoclonal Anti-Estrogen Receptor Antibody in the Immunohistochemical Evaluation of Human Tumors. Cancer Res..

[48] Papadaki C., Mavroudis D., Trypaki M., Koutsopoulos A., Stathopoulos E., Hatzidaki D., Tsakalaki E., Georgoulias V., Souglakos J. (2009). Tumoral expression of TXR1 and TSP1 predicts overall survival of patients with lung adenocarcinoma treated with first-line docetaxel-gemcitabine regimen. Clin. Cancer Res..

[49] Papadaki C., Sfakianaki M., Lagoudaki E., Giagkas G., Ioannidis G., Trypaki M., Tsakalaki E., Voutsina A., Koutsopoulos A., Mavroudis D. (2014). PKM2 as a biomarker for chemosensitivity to front-line platinum-based chemotherapy in patients with metastatic non-small-cell lung cancer. Br. J. Cancer.

[50] Saridaki Z., Tzardi M., Papadaki C., Sfakianaki M., Pega F., Kalikaki A., Tsakalaki E., Trypaki M., Messaritakis I., Stathopoulos E. (2011). Impact of KRAS, BRAF, PIK3CA mutations, PTEN, AREG, EREG expression and skin rash in >/= 2 line cetuximab-based therapy of colorectal cancer patients. PLoS ONE.

